# Suppressive Effect of Hydroquinone, a Benzene Metabolite, on In Vitro Inflammatory Responses Mediated by Macrophages, Monocytes, and Lymphocytes

**DOI:** 10.1155/2008/298010

**Published:** 2009-01-14

**Authors:** Jae Youl Cho

**Affiliations:** School of Bioscience and Biotechnology and the Institute of Bioscience and Biotechnology, Kangwon National University, Chuncheon 200-701, South Korea

## Abstract

We investigated the inhibitory effects of hydroquinone on cytokine release, phagocytosis, NO production, ROS generation, cell-cell/cell fibronectin adhesion, and lymphocyte proliferation. We found that hydroquinone suppressed the production of proinflammatory cytokines [tumor necrosis factor (TNF)-*α*, interleukin (IL)-1*β*, and IL-6], secretion of toxic molecules [nitric oxide (NO) and reactive oxygen species (ROS)], phagocytic uptake of FITC-labeled dextran, upregulation of costimulatory molecules, U937 cell-cell adhesion induced by CD18 and CD29, and the proliferation of lymphocytes from the bone marrow and spleen. Considering that (1) environmental chemical stressors reduce the immune response of chronic cigarette smokers and children against bacterial and viral infections and that (2) workers in petroleum factories are at higher risk for cancer, our data suggest that hydroquinone might pathologically inhibit inflammatory responses mediated by monocytes, macrophages, and lymphocytes.

## 1. INTRODUCTION

Monocytes, macrophages, and lymphocytes are the major types of cells that mediate innate and adaptive immunity. Upon stimulation by bacterial, fungal, or viral infections, macrophages at the infection site release many different proinflammatory cytokines [e.g., tumor necrosis factor (TNF)-*α* and interleukin (IL)-1] and cytotoxic and inflammatory molecules [e.g., nitric oxide (NO) and oxygen species intermediates (ROS)] via the activation of the pattern recognition receptor (PRR). Monocytes continuously migrate to the inflamed site via increased adhesion events mediated by interactions with adhesion molecules such as selectins and integrins. The migrating monocytes eventually differentiate into macrophages for activation and further mediation of inflammatory responses. Macrophages that have been fully activated by bacterial or fungal cell products such as LPS, peptidoglycan (PEG), or *β*-glucan upregulate surface expression of costimulatory molecules such as CD80, CD86, and major histocompatibility molecule class II in order to enhance T cell proliferation. Activated T cells also differentiate into Th1 or Th2 cells to modulate B cell function for antibody production.

Recent decades have seen increased prevalence of several immunopathological diseases including cancers, allergic diseases such as eczema and atopic dermatitis, and respiratory tract infections in chronic cigarette smokers and children [1]. It is thought that multiple environmental and genetic factors, in addition to smoking, are the main reasons for these phenomena. Numerous chemically toxic molecules are particularly known to have pathological effects. Benzene is known to be a human carcinogen [2]. Even though it is chemically hazardous, occupational and habitual exposure to benzene still occurs in the petrochemical and petroleum refining industry as well as through some foods such as coffee [3].

**Figure 1:**
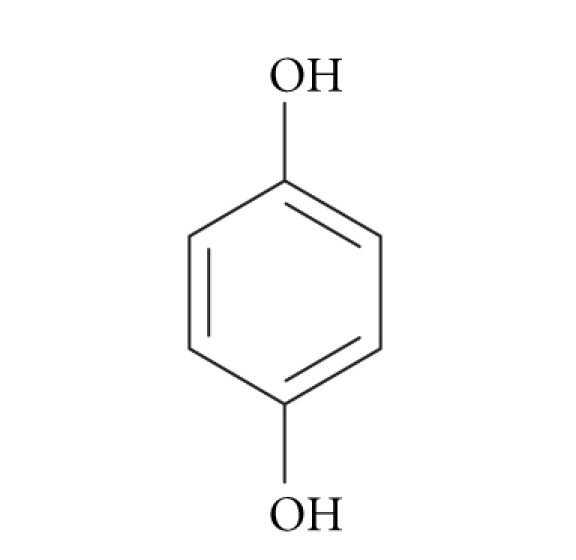
Chemical structure of hydroquinone.

Hydroquinone (benzene-1,4-diol) (Figure 1) is a major component of cigarette smoke and coffee, and a metabolite of benzene via a cytochrome p450-mediated pathway. It is also a heavily used industrial chemical, a petroleum by-product, and an ubiquitous environmental pollutant. Evidence suggesting that cigarette smoking is allergenic and that benzene metabolites are strong haptens [4] stimulated our interest in hydroquinone as an immunotoxicological agent in allergic diseases. Previous reports have suggested that hydroquinone enhances Th2 response-mediated allergic diseases via blockade of interferon (IFN)-*γ* production in Th1 cells, enhanced interleukin-4 production in CD4+ T cells, increased immunoglobulin E levels in antigen-primed mice, and blockade of IL-12 production via suppression of nuclear factor (NF)-*κ*B binding activity [5]. In addition, hydroquinone-mediated inhibition of the production of several cytokines and toxic molecules such as IL-1*β*, IL-2, and NO [6–8] suggests that benzene metabolites might interrupt global immune responses in addition to inducing allergic disease. To date, the pathological importance and mechanisms of benzene metabolites in the modulation of immune responses remain unclear. In the present study, we, therefore, investigated the effect of hydroquinone, a representative reactive benzene metabolite, on the modulation of inflammatory processes mediated by monocytes, macrophages, and lymphocytes.

## 2. MATERIALS AND METHODS

### 2.1. Materials

L-cysteine, phorbol 12-myristate 13-acetate (PMA), curcumin, 1,4-dithiothreitol (DTT), *α*-tocopherol, dihydrorhodamine 123 (DHR123), peptidoglycan (PGN), zymosan, lipopolysaccharide (LPS), *α*-tocopherol, sodium nitroprusside (SNP), concanavalin (Con) A, fluorescein isothiocyanate (FITC)-label dextran, and hydroquinone were obtained from Sigma Chemical Co. (St. Louis, Mo, USA). BAY 11-7082 (BAY) was from Calbiochem (La Jolla, Calif, USA). RAW264.7 and U937 cells were purchased from ATCC (Rockville, Md, USA). All other chemicals were of reagent grade. Aggregation-inducing antibody against CD29 (MEM 101A, purified IgG1) was used as reported previously [9–11]. FITC-labeled antibodies to CD80, CD86, CD29, and CD18 were obtained from Immunotech (Marseilles, France).

### 2.2. Animals

C57BL/6 male mice (6–8 weeks old, 17–21 g) were obtained from DAEHAN BIOLINK (Chungbuk, Korea). The mice were maintained in plastic cages under conventional conditions. Water and pelleted diets (Samyang, Daejeon, Korea) were supplied ad libitum. Studies were performed in accordance with guidelines established by the Kangwon University Institutional Animal Care and Use Committee.

### 2.3. Preparation of peritoneal macrophages

Peritoneal exudates were obtained from C57BL/6 male mice (7-8 weeks old, 17–21 g) by lavage four days after intraperitoneal injection of 1 mL sterile 4% thioglycollate broth (Difco Laboratories, Detroit, Mich, USA). Cells were washed and resuspended in RPMI 1640 containing 2% FBS and plated in 100 mm tissues culture dishes for 4 hours at 37°C in a 5% CO_2_ humidified atmosphere.

### 2.4. Cell culture

RAW 264.7 and U937 cells obtained from American Type Culture Collection (Rockville, Md, USA) were cultured with RPMI medium supplemented with 10% heat-inactivated fetal bovine serum (Gibco, Grand Island, NY, USA), glutamine, and antibiotics (penicillin and streptomycin) at 37°C with 5% CO_2_.

### 2.5. Cytokine production

The inhibitory effect of hydroquinone on TNF-*α* production was determined as previously described [12]. Hydroquinone was solubilized with vehicle (100% ethanol) and diluted with RPMI1640. RAW264.7 cells (2 × 10^6^ cells/mL) were incubated with LPS (2.5 *μ*g/mL) in the presence or absence of hydroquinone for 6 hours. Supernatants were assayed for the levels of TNF-*α*, IL-1*β*, and IL-6 using a mouse TNF-*α* ELISA kit (Amersham, Little Chalfont, Buckinghamshire, UK).

### 2.6. NO assay

RAW 264.7 cells were preincubated with or without tested compounds (hydroquinone and L-NAME) for 30 minutes and continuously activated with LPS (2.5 *μ*g/mL) for 24 hours. Nitrite determination was carried out using Griess reagent [13]. The absorbance of the product dye was measured at 540 nm using a flow-through spectrophotometer.

### 2.7. MTT assay

Cell proliferation was measured by 3-(4,5-dimethylthiazol-2-yl)-2,5-diphenyl tetrazolium bromide (MTT) assay, as described previously [12].

### 2.8. Extraction of total RNA and semiquantitative RT-PCR amplification

The total RNA from LPS-treated RAW264.7 cells was prepared by adding TRIzol reagent (Gibco BRL), according to the manufacturer's protocol. Semiquantitative RT reactions were conducted using MuLV reverse transcriptase as reported previously [14]. Primers (Table 1) (Bioneer, Daejeon, Korea) were used as previously reported [15].

### 2.9. ROS determination

The level of intracellular ROS was determined by the change in fluorescence resulting from oxidation of the fluorescent probe DHR123 [16]. Briefly, 5 × 10^5^ cells/well were incubated with hydroquinone for 30 minutes and then with SNP (125 *μ*M) for an additional 6 hours. After a final incubation with 2.5 *μ*M DHR123 for 1 hour, the intracellular ROS level was determined using flow cytometry.

### 2.10. Phagocytic uptake

To measure the phagocytic activity of RAW264.7 cells, a previously reported method was used with slight modifications [17]. RAW 264.7 cells (1 × 10^6^ cells/mL) were preincubated with or without hydroquinone for 30 minutes, and further incubated for 6 hours. Finally, the cells were further incubated with FITC-dextran (1 mg/mL) for 30 minutes at 37°C. The incubation was stopped by addition of 2 mL of ice-cold PBS, and the cells were washed four times with cold PBS. After fixing the cells with 3.7% formaldehyde, phagocytic uptake was analyzed using a FACScan device (Becton Dickinson, San Jose, Calif, USA).

### 2.11. Cell-cell or cell-extracellular matrix protein (fibronectin) adhesion assay

U937 cell adhesion assay was performed as previously reported [9, 18]. Briefly, U937 cells maintained in complete RPMI1640 medium (supplemented with 100 U/mL of penicillin and 100 *μ*g/mL of streptomycin, and 10% FBS) were preincubated with hydroquinone for 1 hour at 37°C and further incubated with activating (agonistic) antibodies (1 *μ*g/mL) in a 96-well plate. After a 3-hour incubation, cell-cell clusters were determined by homotypic cell-cell adhesion assay using a hemocytometer [18] and analyzed with an inverted light microscope equipped with a COHU high-performance CCD (Diavert) video camera. For the cell-fibronectin adhesion assay, hydroquinone-treated U937 cells (5 × 10^5^ cells/well) were seeded on a fibronectin (50 *μ*g/mL)-coated plate and incubated for 3 hours [19]. After removal of unbound cells with PBS, attached cells were treated with 0.1% crystal violet for 15 minutes. The OD value at 540 nm was measured by a Spectramax 250 microplate reader.

### 2.12. Flow cytometric analysis

Surface levels of CD80, CD86, CD29, and CD18 on U937 or RAW264.7 cells were determined by flow cytometric analysis as reported previously [18]. Stained cells were analyzed on a FACScan device (Becton Dickinson, San Jose, Calif, USA).

### 2.13. Preparation of lymphocytes from bone marrow and spleen and their proliferation assay

Splenocytes or bone marrow-derived cells (5 × 10^5^ cells/well) were prepared from the spleens or bone marrow of mice killed by cervical dislocation under sterile conditions, as described previously [12]. Briefly, the splenocytes or bone marrow-derived cells were released by teasing them into RPMI-1640 medium supplemented with 20 mM N-[2-hydroxyethyl]piperazine-N′-[2-ethanesulfonic acid] (HEPES) buffer. The splenocytes or bone marrow-derived cells (5 × 10^6^ cells/mL) were cultured in 96-well plates in the presence and absence of T or B lymphocyte mitogens (concanavalin A (Con A) and LPS) with hydroquinone in a total volume of 200 *μ*L/well under the same conditions for 48 hours. Proliferation was determined by MTT assay.

### 2.14. Statistic analysis

Student's *t*-test and one-way ANOVA were used to determine the statistical significance between experimental and control groups. *P* values of 0.05 or less were considered to be statistically significant.

## 3. RESULTS

### 3.1. Effect of hydroquinone on macrophage activation

**Figure 2:**
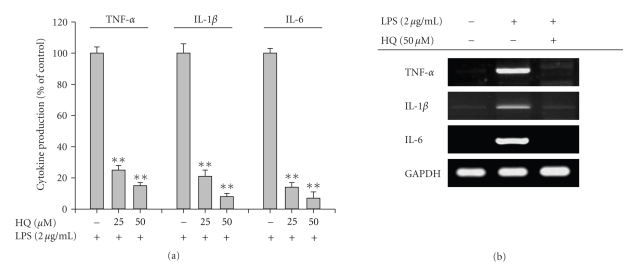
Effect of hydroquinone on the production of cytokines and their mRNA expression in LPS-activated RAW264.7 cells. (a) RAW264.7 cells (2 × 10^6^ cells/mL) were incubated with indicated concentrations of HQ (hydroquinone) in the presence or absence of LPS (2 *μ*g/mL) for 12 hours. Secreted levels of TNF-*α*, IL-1*β*, and IL-6 in culture supernatant were determined by ELISA. (b) The mRNA levels of proinflammatory cytokines from the RAW264.7 cells were determined by semiquantitative RT-PCR. The results show one representative experiment of three. *p < .05 and **p < .01 represent significant difference as compared to control.

Previously, we reported that hydroquinone strongly diminishes LPS-induced TNF-*α* production with an IC_50_ value of 14.8 *μ*M. In this study, we examined its inhibitory effect on other inflammatory cytokines including IL-1*β* and IL-6. Figure 2(a) shows that hydroquinone suppressed production of cytokines without altering cell viability (data not shown). The inhibition of these cytokines also appeared at the transcriptional level (Figure 2(b)), as we had previously shown for TNF-*α* production.

**Figure 3:**
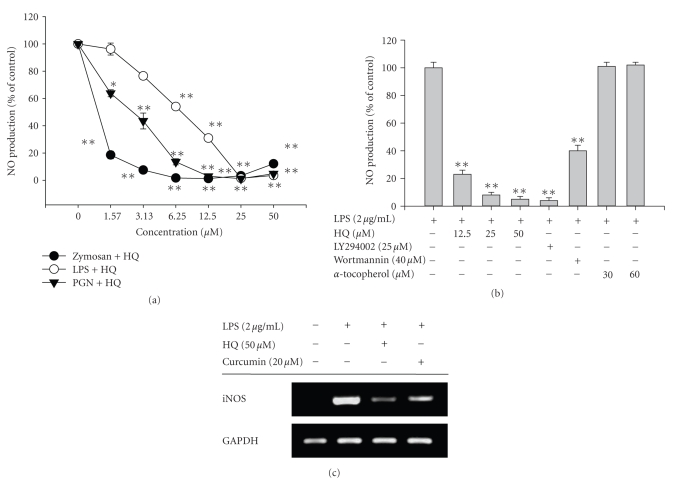
Effects of hydroquinone on the production of NO in LPS-treated RAW 264.7 cells or peritoneal macrophages. (a) and (b) RAW264.7 cells or peritoneal macrophages (1 × 10^6^) were pretreated with various concentrations of HQ (hydroquinone) in the presence or absence of LPS (2 *μ*g/mL), PGN (10 *μ*g/mL), or zymosan (400 *μ*g/mL) for 24 hours. The level of NO was determined by Griess reagent as described in Section 2. (c) The mRNA levels of proinflammatory cytokines from RAW264.7 cells were determined by semiquantitative RT-PCR. The results show one representative experiment of three. *p < .05 and **p < .01 represent significant difference as compared to control.

Since we had also previously reported that hydroquinone could modulate NO production, we next characterized hydroquinone inhibition of NO production under various stimulation conditions. Figure 3(a) shows that hydroquinone inhibited zymosan-induced NO production, whereas LPS-induced NO production was only weakly blocked, suggesting that dectin-1 and TLR2-mediated NO production pathways might be more sensitive targets of hydroquinone. Furthermore, hydroquinone-mediated inhibition of peritoneal macrophage-mediated NO production was also observed (Figure 3(b)). Thus, hydroquinone (as well as the PI3K inhibitors LY294002 and wortmannin) concentration-dependently suppressed NO production in LPS-treated macrophages, whereas the antioxidant *α*-tocopherol did not affect NO production, even under effective antioxidative conditions. Moreover, hydroquinone also diminished LPS-induced iNOS expression (Figure 3(c)), as reported previously [8].

**Figure 4:**
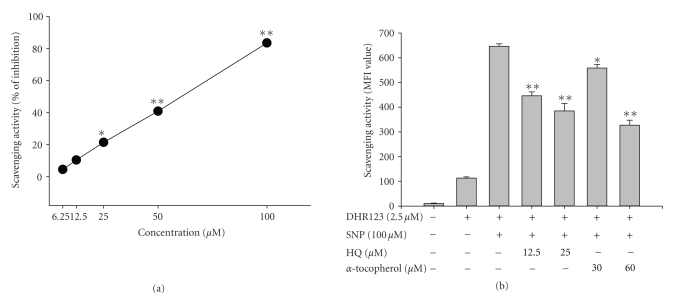
Effects of hydroquinone on ROS generation in SNP-induced RAW 264.7 cells. (a) Antioxidant effect of hydroquinone was examined by DPPH assay as described in Section 2. (b) RAW264.7 cells (1 × 10^6^) were pretreated with various concentrations of HQ (hydroquinone) in the presence or absence of SNP (100 *μ*M) for 30 minutes. The level of generated ROS was determined by flowcytometric analysis as described in Section 2 . *p < .05 and **p < .01 represent significant difference as compared to control.

ROS generation is an important event in inflammatory responses. We, therefore, examined the ROS scavenging activity of hydroquinone. As reported previously, hydroquinone effectively scavenged radical activity as determined by DPPH assay (Figure 4(a)). Furthermore, this compound also neutralized radical generation in SNP-treated RAW264.7 cells, as was the case for *α*-tocopherol (Figure 4(b)).

**Figure 5:**
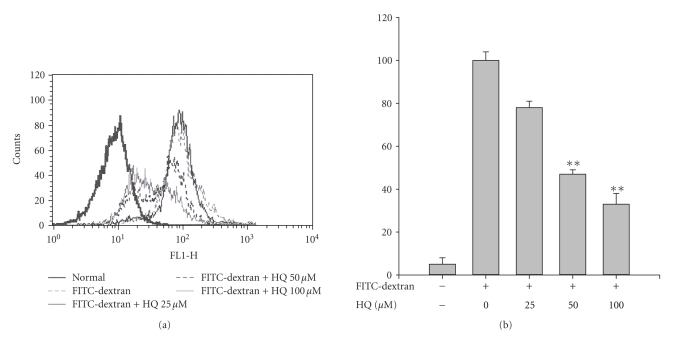
Effect of hydroquinone on the phagocytic uptake of FITC-labeled dextran. RAW264.7 cells (1 × 10^6^) were incubated with in the indicated concentrations of HQ (hydroquinone) in the presence or absence of 1 mg/mL of FITC-labeled dextran for 30 minutes. The uptake of dextran was detected by flow cytometric analysis as described in Section 2. **p < .01 represents significant difference as compared to control.

**Figure 6:**
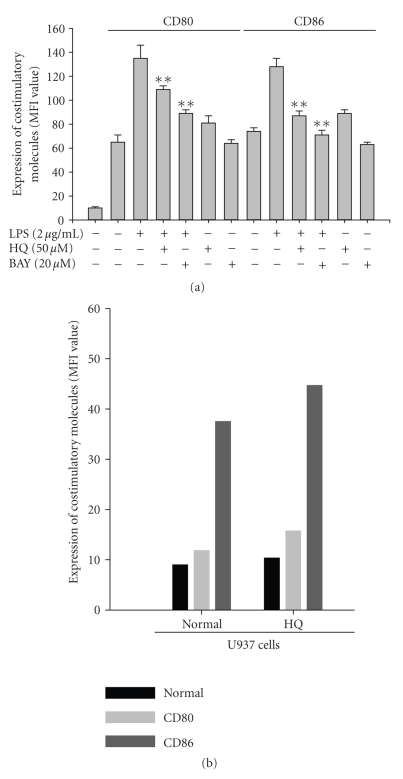
Effect of hydroquinone on surface expression of costimulatory molecules. (a) and (b) RAW264.7 or U937 cells (1 × 10^6^) were incubated with indicated concentrations of HQ (hydroquinone) in the presence or absence of LPS (2 *μ*g/mL) for 24 hours. The surface expression of CD80 and CD86 was determined by flow cytometric analysis as described in Section 2. **p < .01 represents significant difference as compared to control.

Since phagocytosis is representative of inflammatory responses, the effects of hydroquinone on phagocytic uptake of FITC-dextran were investigated. Figure 5 shows that hydroquinone concentration-dependently suppressed dextran uptake, suggesting that it could block the initial response of macrophage activation. Figure 6 shows that hydroquinone and BAY 11-7082 suppressed the upregulation of CD80 and CD86 by LPS-induced dextran uptake. However, hydroquinone did not block normal expression of CD80 and CD86 and rather enhanced it in both RAW264.7 (Figure 6(a)) and U937 cells (Figure 6(b)).

**Figure 7:**
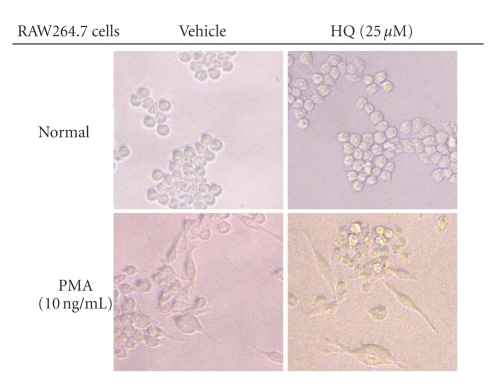
Effect of hydroquinone on PMA-induced morphological changes. RAW264.7 cells (1 × 10^5^) were incubated with indicated concentrations of HQ (hydroquinone) in the presence or absence of PMA (10 ng/mL) for 72 hours. The images of the cells in culture were obtained using an inverted phase contrast microscope attached to a video camera.

Figure 7 shows that 25 *μ*M hydroquinone failed to suppress morphological changes of RAW264.7 cells induced by PMA-mediated actin cytoskeleton rearrangement. These data suggest that the pharmacological effects of hydroquinone might occur in a signal-dependent manner.

### 3.2. Effect of hydroquinone on the cell adhesion of monocytes

**Figure 8:**
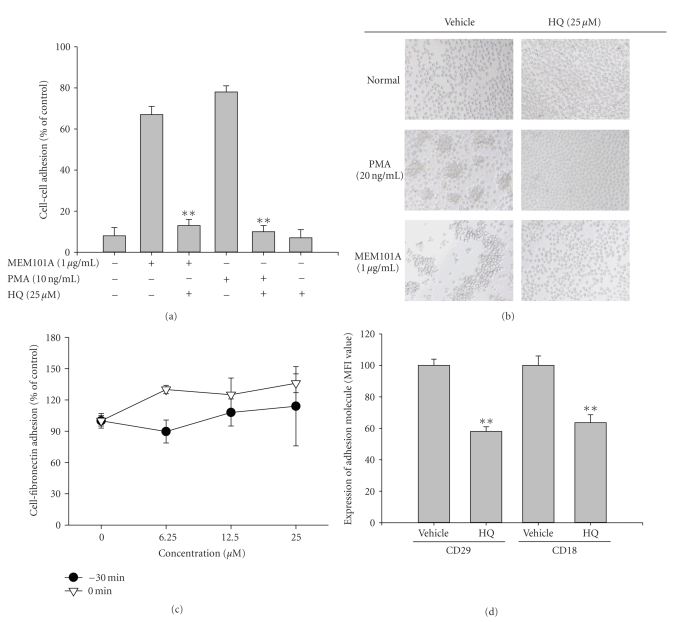
Effect of hydroquinone on cell-cell or cell-fibronectin adhesion events and surface levels of integrins (CD18 and CD29). (a) and (b) U937 cells (1 × 10^6^) were incubated with indicated concentrations of HQ (hydroquinone) in the presence or absence of a proaggregation (activating) antibody against CD29 (MEM101A (1 *μ*g/mL)) for 2 hours. The extent of cell-cell aggregation was determined by quantitative cell-cell adhesion assay as described in Materials and Methods. (b) Images of cells in culture were obtained using an inverted phase contrast microscope attached to a video camera. (c) U937 cells pretreated with hydroquinone were seeded on fibronectin (50 *μ*g/mL)-coated plates and further incubated for 3 hours. Attached cells were determined by crystal violet assay, as described in Section 2. (d) U937 cells were incubated with hydroquinone (12.5 *μ*M) for 3 hours. The surface expression of adhesion molecules (CD18 and CD29) was determined by flow cytometric analysis as described in Section 2. **p < .01 represents significant difference as compared to control.

Since we previously reported the effects of hydroquinone on CD29-mediated cell-cell adhesion, in this study we evaluated fibronectin-cell adhesion and CD18-mediated cell-cell aggregation. Figures 8(a) and 8(b) show that CD18-mediated cell-cell adhesion induced by PMA and CD29-mediated cell-cell adhesion was strongly suppressed by hydroquinone treatment. In contrast, hydroquinone did not block cell-fibronectin adhesion events (Figure 8(c)), indicating that hydroquinone might specifically modulate cell-cell adhesion events and not cell-matrix adhesion. Because these adhesion models are known to depend on CD29 and CD18, the effects of hydroquinone on surface expression of these adhesion molecules were additionally examined. Figure 8(d) shows that hydroquinone treatment significantly diminished surface levels of CD29 and CD18; such downregulation might comprise an inhibitory mechanism for monocyte adhesion.

### 3.3. Effect of hydroquinone on the proliferation of lymphocytes from bone marrow or spleen

**Figure 9:**
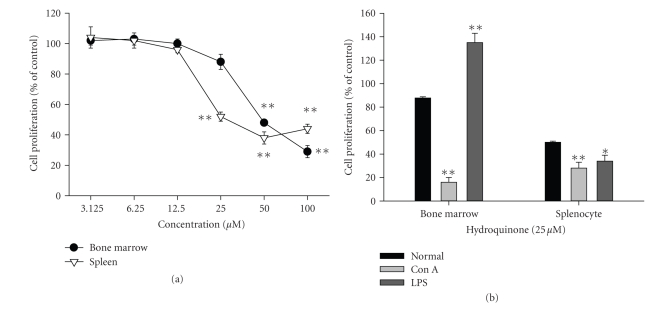
Effect of hydroquinone on the viablility or proliferation of splenic and bone marrow-derived lymphocytes induced by Con A and LPS. (a) Splenic lymphocytes (5 × 10^6^ cells/mL) were incubated with hydroquinone under normal culture conditions. (b) Bone marrow-derived lymphocytes (5 × 10^6^ cells/mL) were incubated with hydroquinone in the presence or absence of Con A (1 *μ*g/mL) and LPS (10 *μ*g/mL). Cell viability or proliferation was determined by MTT assay. *p < .05 and **p < .01 represent significant difference as compared to normal or control.

Lymphocyte proliferation is important for prolonged immune responses. Therefore, we determined whether hydroquinone could modulate lymphocyte proliferation induced by the T cell mitogen Con A or the B cell mitogen LPS. Figure 9(a) shows that hydroquinone concentration-dependently suppressed the normal viability of splenocytes and bone marrow-derived cells. Figure 9(b) shows, however, that T cell and B cell responses to hydroquinone varied according to tissue origin. LPS-induced B cell proliferation from bone marrow-derived cells was not blocked by 25 *μ*M hydroquinone, whereas Con A-induced T cell proliferation was greatly suppressed. In contrast, Con A- and LPS-induced proliferation of T and B cells from splenocytes showed similar levels of inhibition, suggesting that sensitivity of lymphocytes to hydroquinone might be tissue dependent.

### 3.4. Effect of thiol-containing compounds on hydroquinone-mediated inhibition

**Figure 10:**
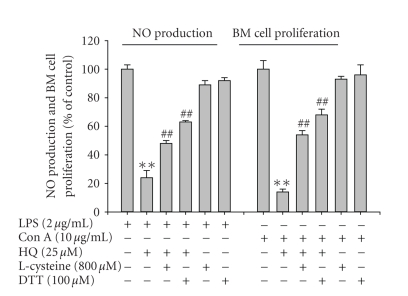
Effect of thiol-containing compounds on hydroquione-mediated inhibition of NO production and lymphocyte proliferation. (a) RAW264.7 cells (1 × 10^6^) were pretreated with L-cysteine (800 *μ*M) or DTT (100 *μ*M) and HQ (hydroquinone, 25 *μ*M) in the presence or absence of LPS (2.5 *μ*g/mL) for 24 hours. The level of NO was determined by Griess reagent as described in Section 2. (b) Bone marrow-derived cells were incubated with L-cysteine (800 *μ*M) or DTT (100 *μ*M) and hydroquinone (25 *μ*M) in the presence or absence of Con A (1 *μ*g/mL) for 24 hours. **p < .01 and ^*##*^ *p* < .01 represent significant difference as compared to control or HQ-treated group.

Finally, the importance of thiolation by hydroquinone was explored using NO production and bone marrow cell proliferation. Interestingly, two thiol-containing compounds, L-cysteine, and DTT abolished the inhibitory effects of hydroquinone on NO production and T cell proliferation from bone marrow-derived cells (Figure 10). These results suggest that hydroquinone inhibition might require thiolation of intracellular target proteins.

## 4. DISCUSSION

In this study, we explored the effect of hydroquinone on inflammatory responses mediated by monocytes, macrophages, and lymphocytes. Our results suggest that hydroquinone can strongly suppress cellular responses required for inflammation, including cytokine production (Figure 2), NO generation (Figure 3), ROS release (Figure 4), phagocytic uptake (Figure 5), surface expression of costimulatory molecules (Figure 6), U937 cell-cell adhesion mediated by CD29 and CD18 (Figure 8), and the proliferation of lymphocytes isolated from bone marrow and spleen (Figure 9).

The molecular mechanism of hydroquinone-mediated downregulation of cellular inflammatory responses is not yet fully understood. Recently, we and others have reported that hydroquinone can reduce the DNA binding of NF-*κ*B [5, 20] and block the phosphorylation of Akt [8]. Although hydroquinone has been reported as a good NF-*κ*B inhibitor, several lines of evidence support that it might block upstream signaling of either NF-*κ*B activation or innate and adaptive immune responses. In the present study, the maximum anti-TNF-*α* effect of hydroquinone was observed when treatment occurred before LPS administration; this suggests that early signaling events occurring within 30 minutes might be potential hydroquinone targets [8]. Additionally, hydroquinone treatment still blocked NF-*κ*B-independent cellular responses such as integrin-mediated cell adhesion [21] and phagocytic uptake (Figure 5). Since the activation of PI3K/Akt, but not MAPKs, is necessary for these inflammation-related cellular events as well as for activation of NF-*κ*B [22], it is possible that hydroquinone might inhibit PI3K/Akt. This is supported by a previous study in which hydroquinone diminished the phosphorylation of Akt, and where the PI3K inhibitors LY294002 and wortmannin displayed broad spectrum inhibition of inflammatory responses [8] similar to that observed for hydroquinone in the present study.

Recently, hydroquinone has been reported to be a potent antioxidant with radical scavenging activities [6, 23], and to induce hemeoxygenase-1 expression. [8] similarly to most antioxidants. However, we unexpectedly found that hydroquinone itself was able to enhance radical generation in RAW264.7 cells to similar extent as LPS [8], suggesting a role as a strong pro-oxidant agent with chemical reactivity [24]. Interestingly, we found that hydroquinone reacts with sulfhydryl groups of thiol compounds, such as L-cysteine, DTT, and N-acetyl-L-cysteine (Figure 10). This chemical reactivity appears to depend on the position of hydroxyl groups in the benzene backbone. Hydroxyl groups in the *para* position were confirmed to be the most reactive, since catechol with *ortho* groups and resorcinol with *meta* groups showed reduced inhibitory effects [25]. Similar modes of action were reported for sesquiterpene lactone compounds with reactive *α*-butyro-*γ*-lactone rings, where these involved the direct binding of NF-*κ*B to DNAvia direct thiolation of 165-cysteine residue of the p65 NF-*κ*B subunit [12, 26]. The effects of hydroquinone may thus occur as the result of direct binding to L-cysteine residues critically involved in the activity of target proteins such as PI3K/Akt.

We and other groups have proposed that hydroquinone could have potential curative effects in inflammatory disease [6, 8, 25]. Nonetheless, environmental toxicity is an important consideration since hydroquinone is topically applied for skin whitening. Because of its potential cancer risk, some countries (e.g., France) have banned the use of this compound. The possible adverse effects are thought to arise from its reactivity, as it is considered the most potent DNA-damaging benzene metabolite. Furthermore, the reactivity of hydroquinone is known to be increased in the presence of NO [27].

**Figure 11:**
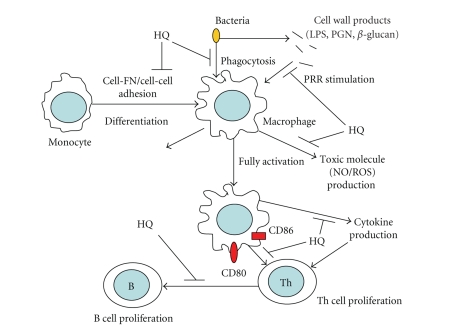
Schematic diagram of hydroquinone-mediated inhibition.

Hydroquinone is also a major component of cigarette smoke and may cause increased rates of higher respiratory tract infection in chronic cigarette smokers [28, 29]. Our results suggest that these immunopathological phenomena might be due to immunosuppressive effects of hydroquinone. In the present study, hydroquinone suppressed most stages of innate immunity mediated by monocytes and macrophages, and of adaptive immunity by lymphocytes, as summarized in Figure 11. Our results suggest that the initial activation of macrophages by bacterial infection might be diminished by hydroquinone exposure. Additionally, hydroquinone might inhibit the migration of monocytes from the blood to infected areas and the aggregation of these cells at infection sites, since it blocked upregulation of integrins (CD18 and CD29) and their functional activation as assessed by homotypic aggregation of monocytic cells. Finally, hydroquinone reduced the normal viability of lymphocytes from bone marrow and spleen as well as their LPS- and Con A-induced proliferation (Figure 9). Previous reports have suggested that inhibition of IFN-*γ* production from activated T cells and IL-12 production from activated macrophages was blocked by hydroquinone treatment and supported an inhibitory role in Th1-type lymphocytes [30–32].

In conclusion, we report that hydroquinone negatively regulates the functional activation of monocytes, macrophages, and lymphocytes (summarized in Figure 11) with respect to proinflammatory cytokine production, secretion of cytotoxic molecules, phagocytic uptake, costimulatory molecule expression, monocytic cell-cell adhesion, and lymphocyte proliferation. These effects appear to be mediated by reactions with thiol groups of L-cysteines in target proteins. Considering that (1) hydroquinone is a major component of cigarette smoke and that (2) immunoprotection of chronic cigarette smokers against lung infections is greatly reduced [29, 33], our data suggest that hydroquinone and other benzene metabolites might play a role in various immunotoxicological conditions.

## Figures and Tables

**Table 1:** Primers used for RT-PCR analysis (F: forward, R: reverse).

Gene		Primer sequences
TNF-*α*	F	5′-TTGACCTCAGCGCTGAGTTG-3′
	R	5′-CCTGTAGCCCACGTCGTAGC-3′
IL-1*β*	F	5′-CAGGATGAGGACATGAGCACC-3′
	R	5′-CTCTGCAGACTCAAACTCCAC-3′
IL-6	F	5′-GTACTCCAGAAGACCAGAGG-3′
	R	5′-TGCTGGTGACAACCACGGCC-3′
iNOS	F	5′-CCCTTCCGAAGTTTCTGGCAGCAGC-3′
	R	5′-GGCTGTCAGAGCCTCGTGGCTTTGG-3′
GAPDH	F	5′-CACTCACGGCAAATTCAACGGCAC-3′
	R	5′-GACTCCACGACATACTCAGCAC-3′
